# Bioprospecting thermophilic glycosyl hydrolases, from hot springs of Himachal Pradesh, for biomass valorization

**DOI:** 10.1186/s13568-018-0690-4

**Published:** 2018-10-15

**Authors:** Sugitha Thankappan, Sujatha Kandasamy, Beslin Joshi, Ksenia N. Sorokina, Oxana P. Taran, Sivakumar Uthandi

**Affiliations:** 1 Department of Agricultural Microbiology, Tamil Nadu Agricultural University, Coimbatore, 641003 India; 2 Boreskov Institute of Catalysis (BIC), Siberian Branch of the Russian Academy of Sciences, Novosibirsk, Russia

**Keywords:** Glycosyl hydrolases, Thermophilic, Biomass, Hot springs

## Abstract

The harnessing of biocatalysts from extreme environment hot spring niche for biomass conversion is significant and promising owing to the special characteristics of extremozymes attributed by intriguing biogeochemistry and extreme conditions of these environments. Hence, in the present study 38 bacterial isolates obtained from hot springs of Manikaran (~ 95 °C), Kalath (~ 50 °C) and Vasist (~ 65 °C) of Himachal Pradesh were screened for glycosyl hydrolases by in situ enrichment technique using lignocellulosic biomass (LCB). Based on their hydrolytic potential 5 isolates were selected and they were *Bacillus tequilensis (*VCB1, VCB2 and VSDB4), and *B. licheniformis* (KBFB2 and KBFB3). Cellulolytic activity assayed by growth under submerged fermentation showed that *B. tequilensis* VCB1 had maximum FPA activity (3.38 IU ml^−1^) in 48 h, while *B. licheniformis* KBFB3 excelled for endoglucanase (EGA of 4.81 IU ml^−1^ in 24 h) and cellobiase (0.71 IU ml^−1^ in 48 h) activities. Among all the thermophilic biocatalysts evaluated, highest exoglucanase (0.06 IU ml^−1^) activity was observed in *B. tequilensis* VSDB4 while endoglucanase of *B. licheniformis* KBFB3 showed optimum specific activity at pH 7 and 70 °C. Further, the presence of *celS, celB and xlnB* genes in the isolates suggest their possible role in biomass conversion. Protein profiling by SDS-PAGE analysis revealed that cellulase isoforms migrated with molecular masses of 75 kDa. The endoglucanase activity of promising strain *B. licheniformis* KBFB3 was enhanced in the presence of Ca^2+^, mercaptoethanol and sodium hypochlorite whereas moderately inhibited by Cu^2+^, Zn^2+^, urea, SDS and H_2_O_2_. The results of this study indicate scope for the possible development of novel biocatalysts with multifunctional thermostable glycosyl hydrolases from hot springs for efficient hydrolysis of the complex lignocellulosic biomass into simple sugars and other derived bioproducts leading to biomass valorization.

**Electronic supplementary material:**

The online version of this article (10.1186/s13568-018-0690-4) contains supplementary material, which is available to authorized users.

## Introduction

Bioprospecting of biomass towards establishment of bio-refineries for augmentation of commodity chemicals and fuel generation warrants mandatory attention. Lignocellulosic (LC) biomass, locally available in plenty, requires less capital investments for bioconversion, reduces greenhouse gas emission and creates employment opportunities in rural livelihood. Almost two-third of the lignocellulosics are comprised of cellulose (40–50%), hemicelluloses (25–35%) and lignin (15–20%). Hydrolytic enzymes are very crucial to break the complex biomass into macromolecules and then into simple sugars, towards the production of biomass-derived sugars, bioproducts and biochemicals. The total cellulase system consists of three enzymatic activities: (i) *Endo*-β-1,4-d-glucanase (EC 3.2.1.4) which randomly cleaves accessible intermolecular β-1,4 glycosidic bonds on the surface of cellulose. (ii) Exoglucanase (CBH, EC 3.2.1.91) that cleaves cellobiose units from both reducing and non-reducing end of cellulose chains. (iii) Finally, β-glucosidases (BG, EC 3.2.1.91) that hydrolyze the remaining glycosidic bonds to form glucose, which can then simultaneously or in a separate step get converted to the desired product by a fermenting biocatalyst.

Multifunctional glycosyl hydrolases (GHs) of microbial origin that possess cellulases, hemicellulases and pectinase activities is gaining importance for bio-based high-value product generation. Bacterial GHs are more stable when compared to fungal hydrolases. Physical heterogeneity of the lignocellulosics and the complexity of the cellulase enzyme system (synergy and/or competition) impose limited substrate surfaces (Hong et al. 2007). It was noted that amidst significant progress that has been made recently towards commercialization of cellulosic ethanol, technological challenges are still remaining as a bottleneck for successful biomass deconstruction (Limayem and Ricke 2012). It is now recognized that cellulose is the rate-limiting substrate in bioethanol production and more efficient enzymes are required to overcome the cellulose recalcitrance to biodegradation. To overcome this impediment, strategies for novel biomass treatment and conversion are the need of the hour for global utilization of lignocellulosic wastes. In this context, one of the strategies could be the use of novel biocatalysts with enhanced stability and improved efficiency for biomass valorization. However, the approach demands a variety of new capabilities which could be only satisfied by microbes from extreme environments.

Thermophilic microbes can produce robust enzyme systems with high hydrolytic potential for cellulose degradation (Zambare et al. 2011). Hence, the discovery of novel thermostable enzymes with enhanced capabilities for biomass deconstruction may lead to significant improvements in integrated biomass processing value chains (Buckley and Wall 2006). The tolerance of high temperatures improves the enzyme robustness and increases the enzyme reaction rates needed for industrial-scale processes thereby decreasing the quantity of enzyme required (Blumer-Schuette et al. 2008). Other added benefits of thermophilic biocatalysts include reduced culture contamination, improved substrate accessibility to enzymes and a reduced viscosity of feedstock allowing the use of higher solid loadings (Kumar and Wyman 2009). Above all, the process of biomass conversion is being operated at a slightly elevated temperature of 50 °C and hence any thermophilic candidate that possesses hyper and multifunctional substrate specificity would be of greater choice. Owing to this perspective, thermophilic cellulase production has been mainly described for thermophilic microorganisms such as *Clostridium* sp. (Bassat and Zeikus 1981), *Thermoascus aurentiacus * (Tong and Cole 1982), *Sporotrichum thermophile*, *Paenibacillus* sp. (Wang et al. 2008), *Brevibacillus* sp. (Liang et al. 2009a), *Anoxybacillus* sp. (Liang et al. 2009b), *Bacillus* and *Geobacillus* (Rastogi et al. 2009). But a cocktail of biocatalysts with simultaneous production of cellulases cum xylanases are of utmost importance and ideal approach towards the simultaneous conversion of the complex cellulose and hemicelluloses to simpler sugars in biorefineries. Whereas only a few reports are available on the use of thermophilic cocktail with cellulase and xylanase for biomass conversion. In this context, the present investigation focuses on the isolation and characterization of thermophiles, from the hot springs of Himachal Pradesh, India for their biocatalytic potential for LC deconstruction using multienzyme systems and augmentation of biorefineries.

## Materials and methods

### Bio-trap enrichment for isolating thermophilic GHs producing bacteria

Thermophilic glycosyl hydrolases (GHs) producing bacteria were isolated by following bio-trap enrichment technique with various natural substrates rich in cellulose, xylan and lignin at the mouth of hot springs (32°01′60N:77°20′60E) of Himachal Pradesh in India (Manikaran (~ 95 °C), Kalath (~ 50 °C) and Vasist (~ 65 °C). Perforated Falcon tubes (15 ml) containing 200–300 mg of substrates (pine needles, para-amino benzoic acid (PABA), vanillin, acid extracted lignin, lignin extracted from black liquor, banana fibre, grapes, paddy straw, corncob and sawdust) were filled with respective thermal water, sealed, and placed in the hot springs for 10 days. The in situ enriched substrates with thermal water from hot springs were used for isolation of thermophilic cellulases and xylanase producing bacteria.

### Isolation and screening of GHs producing thermophilic biocatalysts

Isolation of thermophilic cellulase and xylanase producing bacteria were isolated by dilution plate on Basal medium (KH_2_PO_4_–2.5 g, K_2_HPO_4_–2.5 g, (NH_4_)_2_HPO_4_–1.0 g, MgSO4·7H_2_O–0.2 g, FeSO_4_·7H_2_O–0.01 g, MnSO_4_·7H_2_O–0.007 g for 1 L of media) supplemented with 1% carboxy methyl cellulose (CMC) for cellulolytic and 1% birch wood xylan for xylanolytic bacteria. The plates were incubated at 50 °C till sufficient isolated colonies were observed. The distinct bacterial colonies were observed for morphological and colony characteristics. After incubation, the plates were flooded with 0.1% Congo red followed by destaining with 1 M NaCl (Salem et al. 2008). Positive isolates showed a zone of clearance around the colony and the hydrolytic capacity of the bacterial isolates were calculated as given below,

$$ {\text{Hydrolytic capacity}}\, = \,\frac{\text{Diameter of the zone of clearance}}{\text{Diameter of the colony}} $$

### Identification of bacterial isolates by 16S rDNA sequencing and phylogeny

Genomic DNA extracted from the selected bacterial isolates was used for amplification of 16S rDNA gene sequences using universal primers 27 F (5′-AGAGTTTGATCMTGGCTCAG-3′) and 1492 R (5′-ACGGCTACCTTGTTACGACTT-3′) with the PCR conditions as follows: 95 °C for 5 min; 30 cycles of 94 °C for 1 min, 55 °C for 1 min, and 72 °C for 90 s; and 72 °C for 10 min (Weisberg et al. 1991). PCR product of 1500 bp was resolved by electrophoresis in 1.2% agarose gel in 1 X TAE buffer. Gels were stained with ethidium bromide (10 mg ml^−1^) and visualized on a gel documentation system and gel images were digitalized using a Bio-rad Gel DocXR + system (Hercules, CA, USA). The PCR products were purified using the GeneJET PCR Purification Kit (Thermo Scientific, USA) and were sequenced commercially at BioServe, India and the phylogenetic relationship was analyzed with Mega 7.0.

### Quantification of glycosyl hydrolases (GHs)

The potential thermophilic isolates showing maximum cellulase and xylanase activities on plate assay were further evaluated for quantitative enzyme assays.

### Submerged fermentation process

Initial inoculum for these isolates was developed using LB medium by overnight culture until OD_600_ of 0.6 and then inoculated in a volume of 50 mL of BPS–X production medium (Composition for 100 ml: NaCl 0.5 g, K2HPO4 0.5 g, Peptone 1 g, yeast extract 0.25 g at pH 7.0) supplemented with 1% CMC/xylan for cellulase/xylanase production, respectively (Vincent et al. 2016). The flasks were incubated at 50 °C in an orbital shaker at 110 rpm for 72 h. Enzyme activity was determined at every 24 h intervals in triplicates using analytical grade reagents.

### Preparation of crude enzyme extract

After incubation, the cultures were centrifuged at 5000 rpm, 10 min at 4 °C and the cell-free culture supernatant was used for determination of various enzyme activities.

### Filter paper activity for cellulase (FPA)

The FP activity of the crude enzyme extract was measured according to Zhang et al. (2009). One ml of the crude enzyme was incubated with 2 ml of 0.1 M citrate buffer (pH 4.8) containing 0.05 g Whatman No. 1 filter paper as the substrate. After incubation for 1 h at 50 °C, the reducing sugars in the reaction mixture were determined by the addition of 3 ml of DNS reagent as per the method of Nelson (1944). The tubes were placed in a boiling water bath for 5 min and after cooling, the absorbance of the samples was measured at 540 nm. One unit of FP activity is equivalent to one micromole of glucose liberated per ml of culture filtrate per minute.

### Endoglucanase (CMCase)

Endoglucanase activity of the cell-free culture supernatant was determined according to Zhang et al. (2009). The supernatant containing the enzyme (500 μl) was mixed with 500 μl substrate (1% CMC prepared in 50 mM Sodium phosphate buffer, pH 7) and 500 μl of 50 mM Sodium phosphate buffer (pH 7) and incubated in a water bath at 50 °C for 30 min. After incubation, the reaction was terminated by adding 3 ml of 3, 5-dinitrosalicylic acid (DNS) reagent and the reducing sugars were estimated as previously mentioned. One unit of enzymatic activity is defined as the amount of enzyme that releases 1 μmol of reducing sugars (measured as glucose) per ml per minute.

### Exoglucanase (Avicelase)

Avicelase activity was determined according to Zhang et al. (2009). For exoglucanase assay, 500 μl of crude enzyme was mixed with 500 μl substrate (1.25% Avicel in acetate buffer) and incubated at 50 °C for 2 h. The reaction was terminated by submerging the tubes in an ice-cold water bath and the total soluble sugars were determined by phenol–sulfuric acid method (Dubois et al. 1956). After cooling, the absorbance of the samples was measured at 490 nm and the enzymatic activity of exoglucanase was defined in international units (IU). One unit of enzymatic activity is defined as the amount of enzyme that releases 1 μmol of glucose equivalent per minute from Avicel.

### Aryl β-glucosidase (cellobiase; EC3.2.1.21)

The aryl β-glucosidase was assayed based on chromogenic *o*-nitrophenol release from o-nitrophenyl-β-d-glucopyranoside (Wood and Bhat 1988). The reaction mixture consisted of 1.0 ml of pNPG solution (5 mM in acetate buffer) and 2.0 ml enzyme of appropriate dilution. The reaction was initiated at 50 °C for 15–30 min and stopped by the addition of 40.0 ml of NaOH/glycine buffer (0.4 M, pH 10.8). The absorbance of the liberated *o*-nitrophenol was measured at 425 nm and one unit of activity corresponding to the amount of enzyme capable of liberating 1 pmol o-nitrophenol min^−l^ under assay conditions. Cellobiase was determined by measuring the glucose released, by the method of Nelson (1944).

### PCR screening of isolates for GHs specific genes

The cellulolytic and xylanolytic isolates thus selected were screened for cellulase and xylanase gene using gene-specific primers as given Additional file 1: Table S1 and the amplicons were resolved in 1% agarose gel.

### Partial purification of extracellular cellulases

About 25 ml of the crude enzyme solution was saturated using ammonium sulfate and the mixture was left overnight at − 40 °C for precipitation (Lee et al. 2008). The precipitates were collected by centrifugation and dissolved in 2 ml of 0.1 M phosphate buffer (pH 7.0). The enzyme collected after ammonium sulfate precipitation was dialyzed against 0.1 M phosphate buffer at 4 °C with three changes of buffer. The partially purified protein was assayed for enzyme activity and protein content as mentioned elsewhere.

### Endoglucanase activity on agarose plate

Endoglucanase activity was confirmed on CMC (0.1%) agarose plates as per Zhang et al. (2009). Wells were drilled in CMC agarose plates and 20 μl of the partially purified enzyme was added and incubated at 50 °C for overnight. After incubation, the plates were washed with distilled water, and staining (0.1% congo red solution) and destaining (1 M NaCl) were performed as mentioned above to detect the hydrolytic zone (yellow halo with red background).

### Kinetic studies of endoglucanase

The Michaelis–Menten kinetic model of the single-substrate reaction was investigated for the enzyme produced by the bacterial isolates. The Michaelis–Menten equation is given as follows,

$$ v\, = \,\frac{{{\text{V}}_{ \text{max} } \left[ {\text{S}} \right]}}{{{\text{K}}_{m} + \, \left[ {\text{S}} \right]}} $$

The apparent kinetic parameters (Vmax and Km) of the cellulase were determined by varying the concentration of CMC from 1 to 5% in 50 mM citrate buffer (pH 4.5). The CMC solutions at different concentrations (1–5%) were treated with the purified cellulase and the extent of hydrolysis was investigated. The data were analyzed using Graph Pad Prism software (version 5.0).

### SDS–polyacrylamide gel electrophoresis of partially purified endoglucanase and xylanase

The protein was separated by SDS-PAGE in 12% polyacrylamide gels according to Laemmli (1970). Protein bands were detected by Coomassie-brilliant blue staining solution and destained with glacial acetic acid:methanol: water (5:45:50) and documented using Bio-rad Gel DocXR + system (Hercules, CA, USA).

### Native PAGE and gel diffusion assay

The protein samples from culture supernatants were separated with a 12% Native–polyacrylamide gel electrophoresis at 4 °C. Following this, the gel was placed on an agarose plate containing 0.1% CMC and incubated for 1 h, at 50 °C. The plate was stained with 0.1% congo red for 30 min and finally washed with 1 M NaCl to detect enzyme activity (Zhang et al. 2009).

### Characterization of endoglucanase *of B. licheniformis* KBFB3

#### Protein determination and specific activity

Protein content was determined using Bradford reagent (Biorad) in a multimode microtitre plate reader (SpectraMax@i3x) with bovine serum albumin as standard at wavelength 595 nm. The specific activity of Endoglucanase was calculated and expressed in terms of IU per mg of protein.

#### Effect of temperature and pH on endoglucanase activity

For determination of optimal temperature, the assay was carried out using 0.5% (w/vol) CMC as a substrate in a total volume of 120 µl containing 100 mM Sodium Phosphate (pH 7.0). The assay mixture was incubated at 45–85 °C, at 5 °C intervals for 30 min and the reaction was stopped by boiling for 10 min. The free glucose released was measured as stated above.

The optimal pH for Endoglucanase (endoglucanase) activity was determined in various buffers with different pHs viz., Ammonium citrate buffer (3.0), Sodium acetate (4.5), Sodium Phosphate (7.0), Tris (8.5) and Ammonia buffer (9.5).

#### Effect of metal ions on endoglucanase activity

The effect of various metal ions on partially purified cellulase was determined by the presence of NaCl, KCl, CaCl2, ZnSO4 and CuSO4, each at 5 mM concentration. The enzyme was incubated with different metals at 70 °C for 1 h and assayed under standard assay conditions (Zhang et al. 2009). The residual endoglucanase activity was estimated against the control, in which metal ions were not present.

#### Effect of inhibitors on endoglucanase activity

The partially purified enzyme activity was pre-incubated with different inhibitors like ethylene diamine tetra acetic acid (EDTA), β-mercaptoethanol, dithiothreitol (DTT) and urea at a concentration of 5 mM for 1 h at 70 °C. The residual activity was determined under standard assay conditions.

#### Effect of surfactants and oxidizing agents on endoglucanase activity

The effects of surfactants viz., Triton X 100, Tween-80, SDS (0.5% v/v), and oxidizing agents viz., H_2_O_2_ and sodium hypochlorite (0.5% v/v) were investigated in order to characterize the enzyme. The partially purified enzyme was incubated with the above-mentioned reagents for 1 h at 70 °C and the residual Endoglucanase activity was determined under standard assay conditions against control, in which additives was not present.

## Results

### Isolation and screening of cellulolytic and xylanolytic thermophilic bacteria

#### Screening of bacteria and assay for hydrolytic activity

**Fig. 1:**
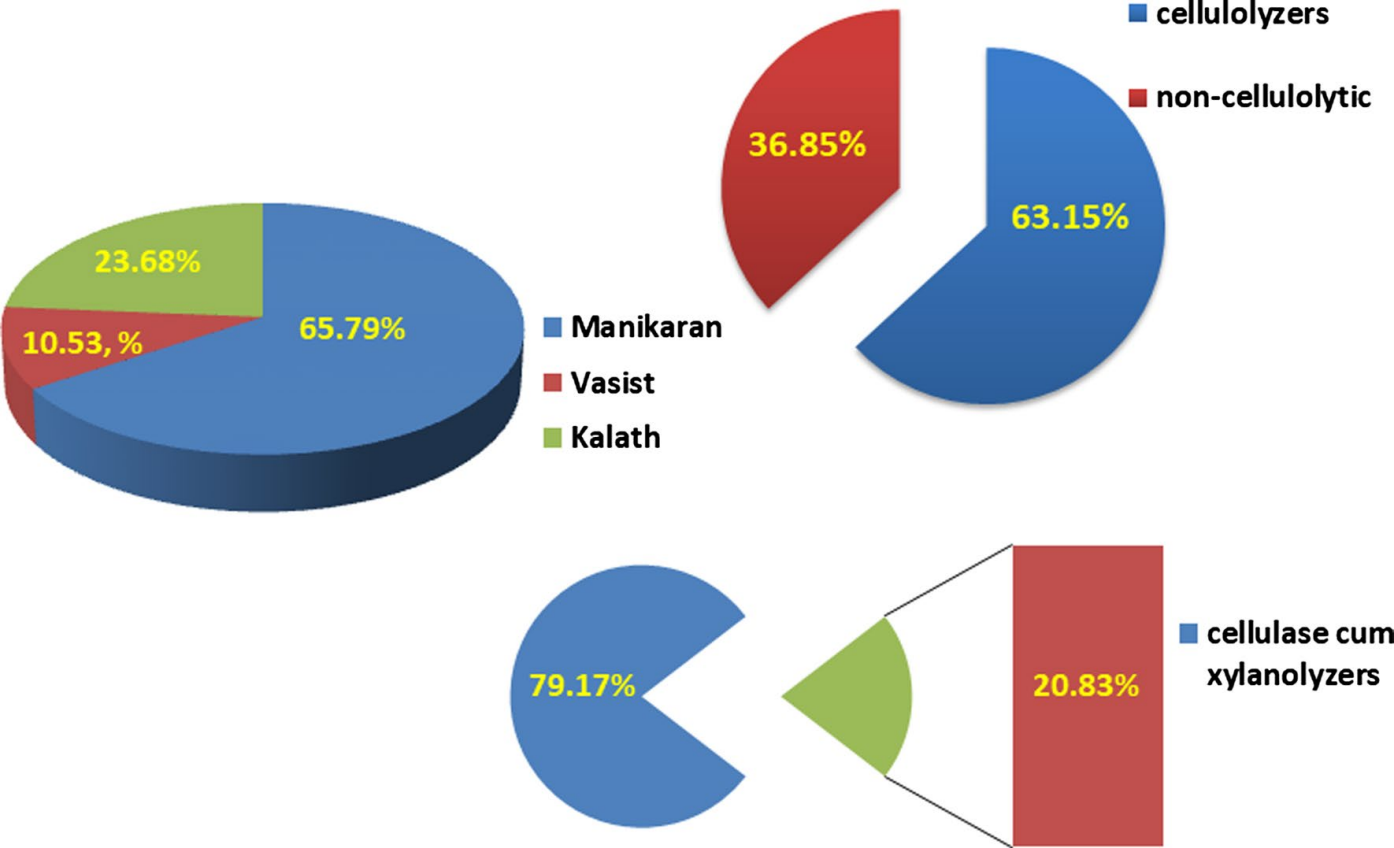
Distribution of isolated thermophiles in hot springs of Himachal Pradesh. Thermophilic bacterial isolates were obtained from three hot springs Vasist, Kalath and Manikaran of Himachal Pradesh, India. The isolates were subjected to qualitative screening for cellulose utilization on CMC agar plates. Based on the hydrolytic capacity they were classified as cellulolytic and non-cellulolytic bacterial isolates

Thermophilic bacteria with cellulolytic and xylanolytic activities were isolated from hot springs (Manikaran (~ 95 °C), Kalath (~ 50 °C) and Vasist (~ 65 °C)) of Himachal Pradesh using from various lignocellulosic substrates viz., pine needles, PABA, vanillin, acid extracted lignin, lignin extracted from black liquor, banana fibre, grapes, paddy straw, crop residue, corncob and sawdust. The enrichment technique employed yielded a total of 38 isolates from the enriched samples, of which 25, 4 and 9 isolates were respectively obtained from samples of Vasist, Kalath and Manikaran (Table 1; Fig. 1).

**Table 1:** Thermophilic bacterial isolates from hot springs isolated by in situ biotrap-enrichment

Substrate and location	Isolates
Vasist	
Vanillin	VVB1, VVB2
Paddy straw	VPSB1, VPSB2
Crop residue	VCRB1, VCRB2, VCRB3
Corn cob	VCB1, VCB2, VCB3
Saw dust	VSDB1, VSDB2, VSDB3, VSDB4
Banana fibre	VBFB1, VBFB2, VBFB3
Pine needle	VPB1, VPB2, VPB3
Para amino benzoic acid	VPAB1
Acid extracted lignin	VAB1
Black liquor	VBLB1, VBLB2, VBLB3
Kalath	
Banana fibre	KBFB1, KBFB2, KBFB3
Grapes	KGB1
Manikaran	
Banana	MBB1, MBB2, MBB3
Banana fibre	MBFB1, MBFB2, MBFB3
Grapes	MGB1, MGB2, MGB3

**Fig. 2:**
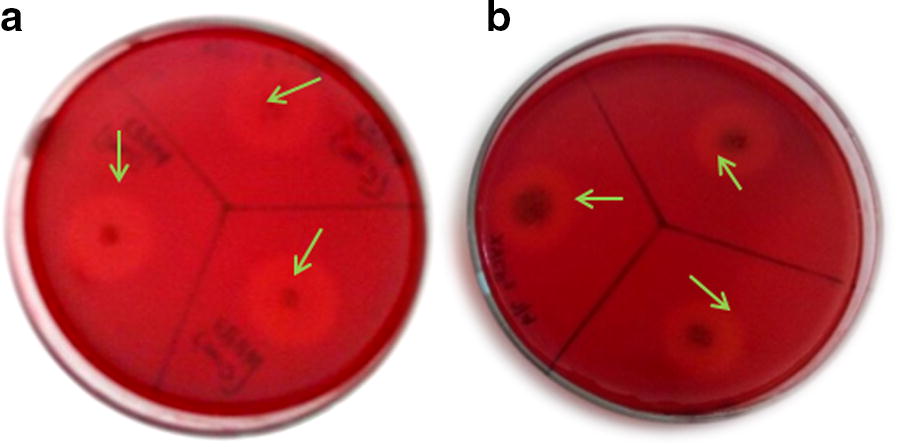
**a** Cellulose and **b** xylan plates showing clearing zone by thermophilic bacterial isolates. The thermophilic bacterial isolates were grown on CMC agar and xylan agar plates respectively and incubated at 50 °C for 48 h. The plates were flooded with 0.1% congored followed by destaining with 1 M NaCl. The presence of a halo around the colonies represents hydrolytic activity, cellulolytic as well as xylanolytic respectively

Active digestion of cellulose and xylan by the thermophilic biocatalysts in congo red plate assays caused a halo clear zone formation indicating endoglucanase and xylanase secretion into the solid medium (Fig. 2). Among the 38 isolates, 24 isolates that showed cellulase activity at ≥ 50 °C were selected for further studies. Of the 24 cellulolytic isolates, 19 were positive for xylanase activity at ≥ 50 °C. The hydrolytic capacity index (HC) of five promising isolates VCB1, VCB2, VSDB4, KBFB3 and KBFB2 for cellulolytic and xylanolytic activities ranged between 1.46 to 4.06 and 1.67 to 5.30 respectively (Table 2). The maximum cellulase activity was observed with KBFB2 (4.06), while xylanase was observed with KBFB3 (5.30) followed by KBFB2 (2.60) and VCB2 (2.18).

**Table 2:** Hydrolytic capacity of the potential bacterial isolates for cellulolytic cum xylanolytic activity

Isolate	Cellulolytic activity	Xylanolytic activity
VCB1	2.20 ± 0.14^a^	2.14 ± 0.13^b^
VCB2	1.93 ± 0.12^a^	2.18 ± 0.14^b^
VSDB4	1.46 ± 0.09^a^	1.67 ± 0.29^b^
KBFB2	*4.06 ± 0.25* ^a^	2.60 ± 0.16^b^
KBFB3	3.91 ± 0.24^a^	*5.30 ± 0.33* ^a^

Values represent mean (± standard error) (n = 3) and the values representing same alphabets are not significant from each other as determined by DMRT at p = 0.05

Maximum values are in italic

### Phylogeny and identification of GHs producing thermophiles

**Fig. 3:**
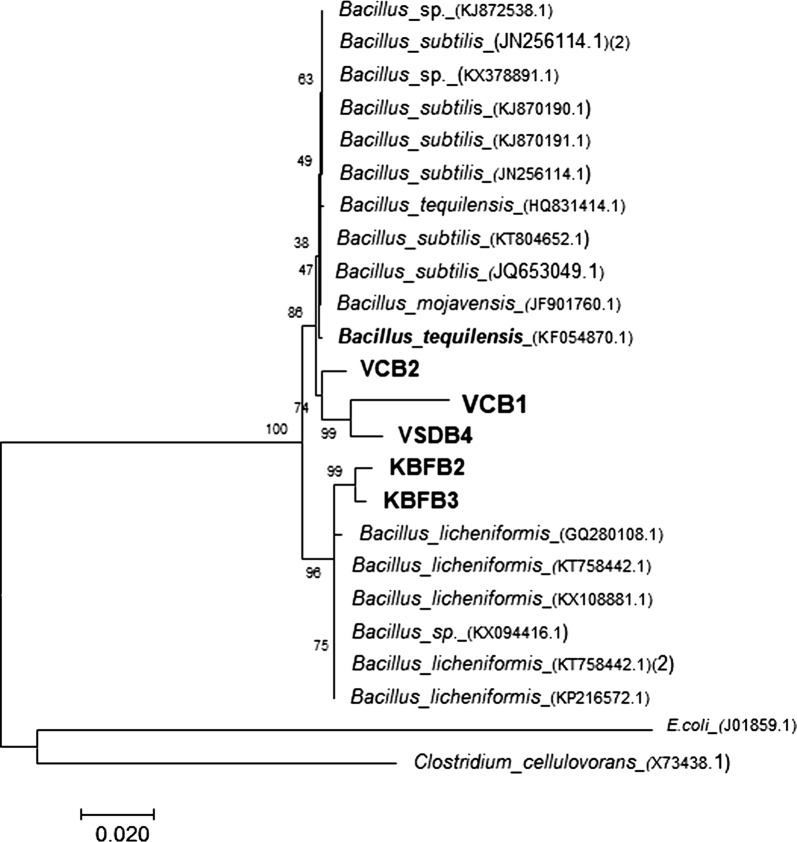
Phylogenetic dendogram based on the 16S rDNA sequences of isolates and reference sequences retrieved from genbank. The phylogenetic tree was constructed based on neighbourhood joining method (NJ) using Mega 7.0 software with a bootstrap value of 0.020

The promising five thermophilic isolates obtained were identified by 16S rDNA gene sequence analysis. The 1.5 kb amplicon obtained through PCR was sequenced and further analyzed by NCBI BLAST nucleotide search and the nearest match from GenBank data was reported (Table 3). The phylogenetic tree constructed on the aligned datasets using neighbour joining (NJ) method using MEGA 7.0 (Fig. 3) revealed that all the isolates obtained from thermal springs were Gram-positive and belong to the phylum *Firmicutes*. The closest phylogenetic neighbours according to the 16S rDNA gene sequence data for the isolates VCB1, VCB2, and VSDB4 were *Bacillus tequilensis* KF054870.1 with 74 and 99% homology, respectively. The isolates, KBFB2 and KBFB3 have a close identity with *Bacillus licheniformis* GQ280108.1 (99 per cent).

**Table 3:** Identification of thermophilic bacterial isolates by 16S rRNA gene sequence homology

Isolate	Sequence homology			Phylum
	Closest species^a^	Accession no.	Per cent homology^b^	
VCB1	*Bacillus tequilensis*	MG098075	74	*Firmicutes*
VCB2	*Bacillus tequilensis*	MG098076	99	*Firmicutes*
VSDB4	*Bacillus tequilensis*	MG027696	99	*Firmicutes*
KBFB2	*Bacillus licheniformis*	MG028595	99	*Firmicutes*
KBFB3	*Bacillus licheniformis*	MG028594	99	*Firmicutes*

^a^Species identified based on 16S rRNA gene sequence similarity

^b^Per cent similarity of the sequence in BLAST analysis

### PCR based screening of potential thermophiles for cellulase and xylanase gene

**Fig. 4:**
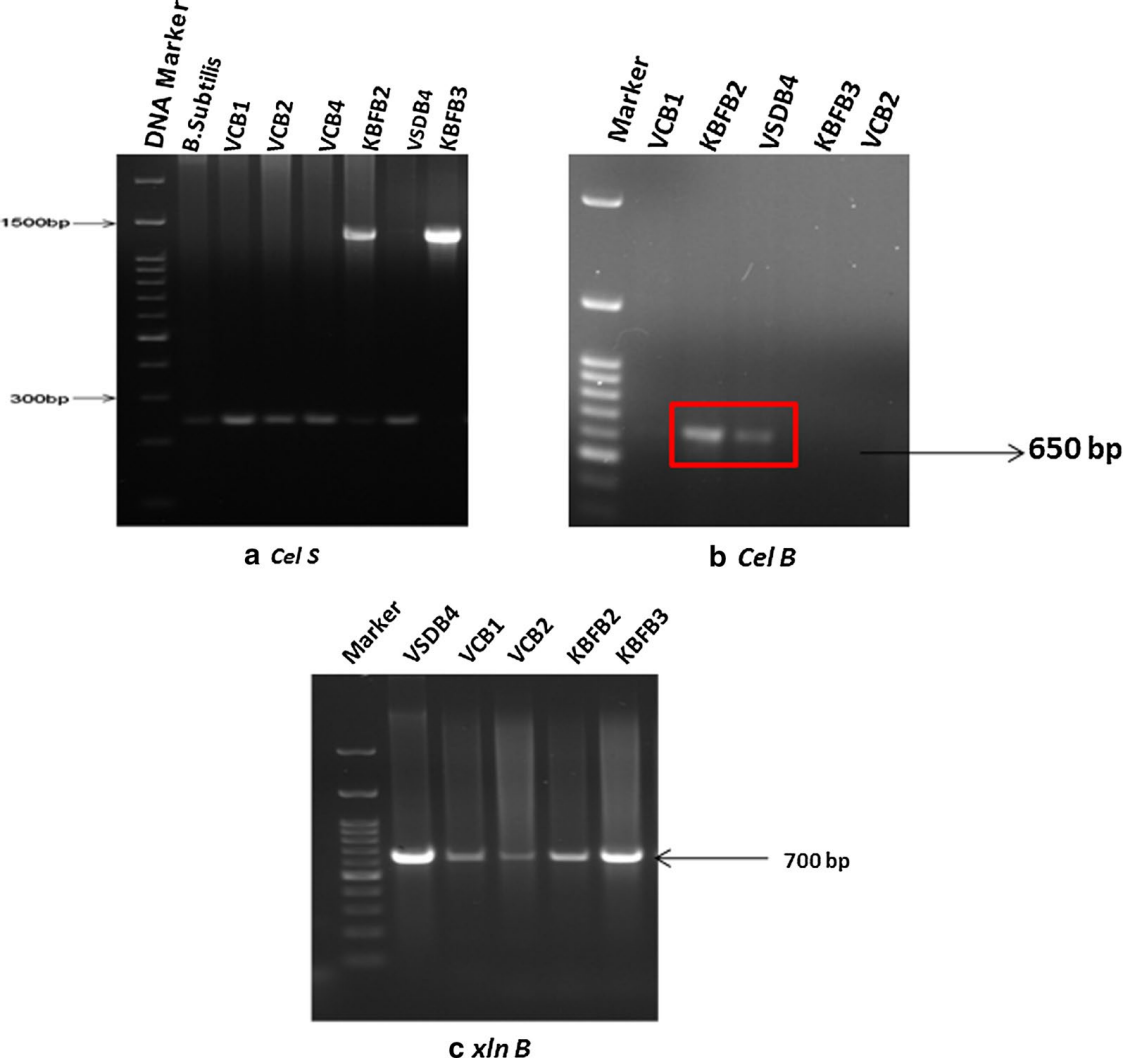
PCR based confirmation of cellulolytic and xylanolytic activity by bacterial isolates. Further, the thermophilic bacterial isolates were confirmed for cellulolytic and xylanolytic activity using specific primers for cellulase (celS), cellulose binding domain (celB) and xylanase (xlnB). **a** *Cel S* **b** *cel B* **c** *xln B*

The thermophilic biocatalysts used for enzyme assay were further confirmed by the presence of cellulase (*celS* and *celB and* xylanase gene (*xln*) using gene-specific primers. Cellulase primer (*CelS*) resulted in the amplification of about 250 bp amplicons in *B. tequilensis* VCB1, *B. tequilensis* VCB2, *B. tequilensis* VSDB4 and *Bacillus subtilis* VCB4 (positive control), while 1300 bp amplicons were observed in *B. licheniformis* KBFB2 and *B. licheniformis* KBFB3 (Fig. 4a). Moreover, amplification of cellulose binding domain resulted in 650 bp amplicon only in two isolates, *B. licheniformis* KBFB2 and *B. licheniformis* KBFB3 (Fig. 4b) whereas, during xylanase screening, 700 bp amplicons were incurred in xylanase positive thermophilic isolates (Fig. 4c). These results confirmed the presence and role of cellulase and xylanase gene in glycosyl hydrolase activities of the thermophilic biocatalysts under study.

### GHs activity of thermophiles

#### FP activity

**Fig. 5:**
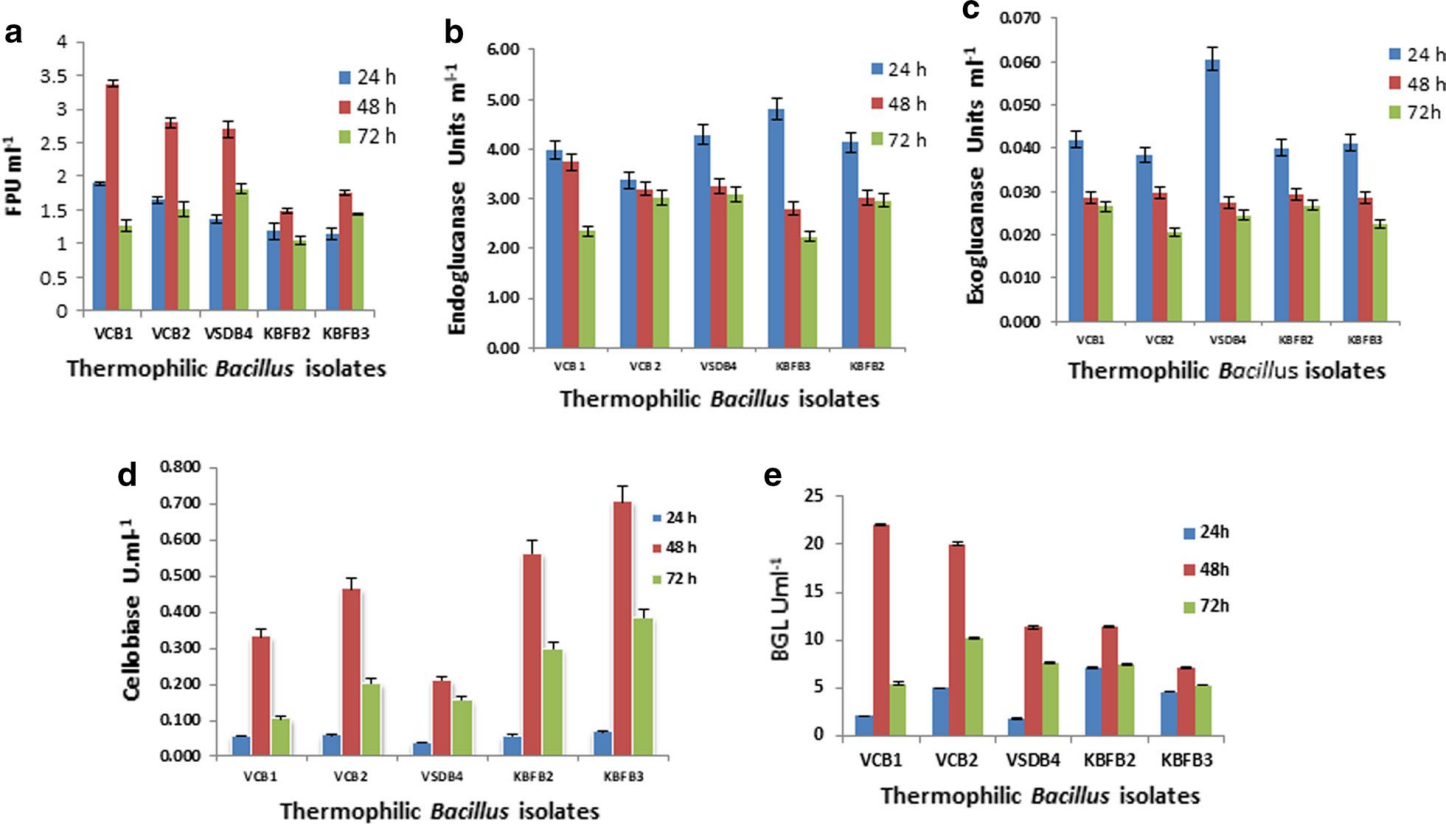
Cellulase activity profile by the bacterial isolates on different substrates. The bacterial isolates were grown on their respective production media with CMC, Avicel and cellobiose. Enzyme activity was assayed by incubating the crude enzyme with various substrates **a** filter paper; **b** CMC; **c** avicel; **d** cellobiose; **e** pNPG, and the released sugars were estimated

FPA represents total cellulase activity based on the ability to utilize filter paper and release reducing sugar units. All the isolates produced a significant amount of FPA until 48 h which declined thereafter. Among the isolates, *B. tequilensis* VCB1 and *B. tequilensis* VCB2 showed maximum activities of 3.38 and 2.8 IU ml^−1^ at 48 h followed by *B. tequilensis* VSDB4 (2.72 IU ml^−1^) (Fig. 5a).

### Endoglucanase (endo-1,4-β-glucanase; carboxymethylcellulase; EC3.2.1.4)

The promising thermophilic isolates were able to produce endoglucanase utilizing CMC as a substrate. A maximum titre of 4.81 IU ml^−1^ was observed with *B. licheniformis* KBFB3 followed by *B. tequilensis* VSDB4 (4.28 IU ml^−1^) at 24 h. The endoglucanase activity of other thermophilic isolates was in the range of 3.02 to 3.97 IU ml^−1^ (Fig. 5b), which however declined gradually later beyond 24 h irrespective of all the isolates.

### Exoglucanase (avicelase)

Maximum exoglucanase activity was observed with *B. tequilensis* VSDB4 (0.061 IU ml^−1^) followed by B*. tequilensis* VCB1 (0.042 IU ml^−1^) and the rest of the isolates produced the same level of activity at 24 h of growth. A drop in the activity was noticed at 48 h in all the isolates and there was no significant difference among the isolates for exoglucanase activity. However, the enzyme activities were found to decline gradually after 24 h (Fig. 5c).

### Aryl β-glucosidase (cellobiase; EC3.2.1.21)

The cellobiase activity of the five thermophilic bacterial isolates grown in production media containing CMC and cellobiose (1%) were assayed using cellobiose and o-nitrophenyl-β-d-glucopyranoside (pNPG) as substrates. The cellobiase activity registered a steady increase from 24 h and reached a maximum at 48 h, although declined thereafter (Fig. 5d, e).

The cellobiase activity for CMC grown isolates was observed to be in the range of 0.210 to 0.705 IU ml^−1^ at 48 h registering a maximum by *B. licheniformis* KBFB3 (0.705 IU ml^−1^) followed by *B. licheniformis* KBFB2 (0.562 IU ml^−1^) and *B. tequilensis* VCB2 (0.464 IU ml^−1^).

On the other hand, β-glucosidase activity on pNPG for cellobiose grown cultures was found to be higher than those grown in medium with CMC. Enzyme activity ranged from 2 to 23 IU ml^−1^ with a maximum titre recorded at 48 h. However, thereafter the enzyme activity recorded a declining trend. Among the isolates, *B. tequilensis* VCB1 registered significant BGL activity of 23 IU ml^−1^ followed by *B. tequilensis* VCB2 (20.4 IU ml^−1^) and *B. licheniformis* KBFB2 (11.44 IU ml^−1^).

### Kinetic analyses of endoglucanase

The apparent kinetic parameters (V_max_ and K_m_) of the cellulase were determined by varying the CMC concentration from 1 to 5% corresponding to 38 to 190 mM in 50 mM sodium citrate buffer (pH 5.0). The Michaelis–Menten plot (Additional file 2: Fig. S1) showed that maximum best fit values of K_m_ and V_max_ for the elite cellulolytic bacterial isolates (*Bacillus* sp.) under the study had fallen in the range of 44.20 to 111.5 m Mol and 5.34 to 19.73 µmol min^−1^, respectively registering maximum K_m_ and V_max_ values by *B. licheniformis* KBFB3 compared to other isolates (Additional file 1: Table S2). Hence, it is evident from the study that the enzyme endoglucanase of *B. licheniformis* KBFB3 showed maximal hydrolytic efficiency for CMC with the highest Km and Vmax values. Hence, for further characterization of endoglucanases, *B. licheniformis* KBFB3 was considered.

### Partial purification and characterization of GHs

**Fig. 6:**
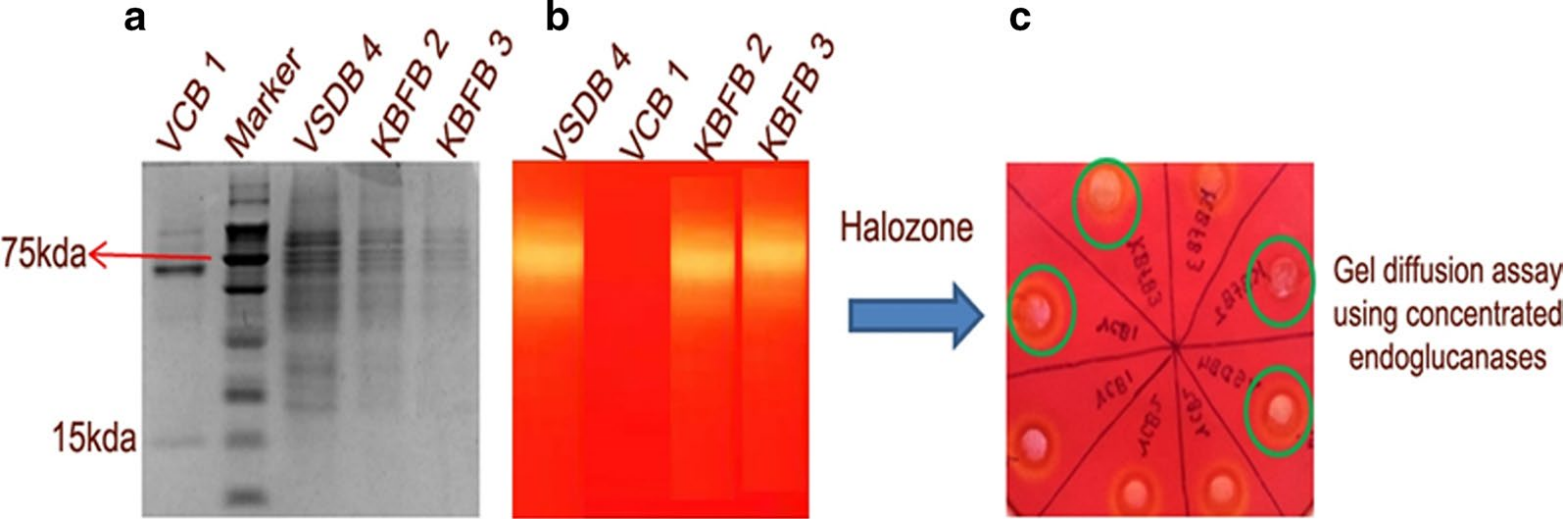
SDS PAGE and zymogram analysis showing cellulolytic activity. **a** SDS profile of endoglucanase by the promising thermophilic isolates grown on culture media with CMC; **b** invitro endoglucanase activity in polyacrylamide gel with CMC; **c** halo zones showing endoglucanase activity on CMC + agarose plates stained with CongoRed

The partially purified cell-free culture supernatant of the selected thermophilic biocatalysts which showed significant titre value for all the cellulases (n = 4) was analyzed by SDS-PAGE (Fig. 6). From Fig. 6a it was inferred that bands corresponding to the cellulase proteins migrated with molecular mass 75 kDa. Zymogram analysis with activity staining for the said isolates was also carried out parallely and the Congo red staining followed by destaining using 1 M NaCl showed clear halo formation (Fig. 6b). In addition, the extracellular endoglucanase on agarose plate with 1% CMC also confirmed the degradation of CMC by forming a yellow colour halo around the well except for VCB1 (Fig. 6c).

The partially purified endoglucanase from *B. licheniformis* KBFB3 had the specific activity of 16.3 U mg^−1^ with 2.34-fold purity (Additional file 1: Table S3). The promising strains *B. tequilensis* VCB1 and *B. licheniformis* KBFB3 were submitted in NAIMCC under the accession no. TB2589 and TB2590 respectively.

### Characterization of purified enzyme

#### Effect of temperature and pH on endoglucanase activity of *B. licheniformis* KBFB3

**Fig. 7:**
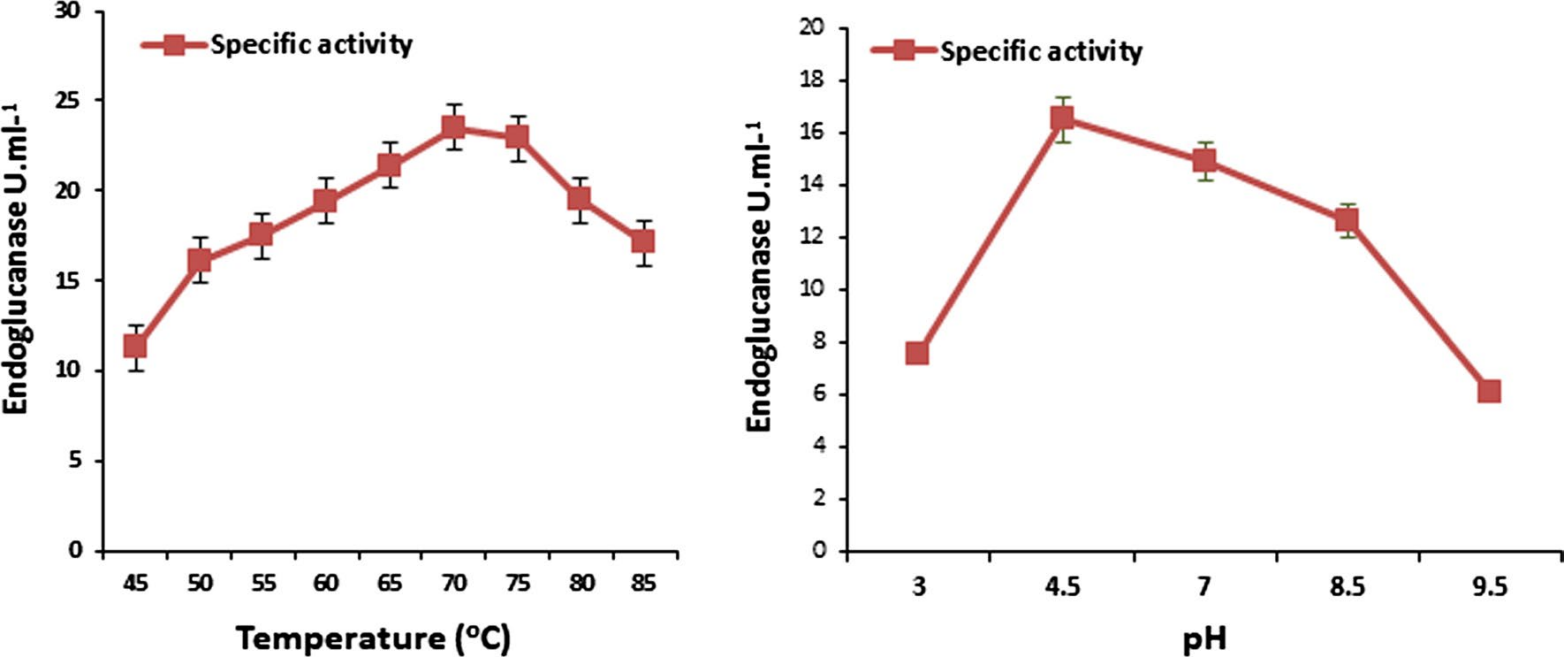
Effect of temperature and pH on endoglucanase activity for the strain *Bacillus licheniformis* KBFB3. The optimum pH and temperature for endoglucanase activity for the best isolate *Bacillus licheniformis* KBFB3 was determined by growing it in production media amended with CMC 1% in the different range of temperature and pH

Profiles of specific enzyme activity obtained using CMC as a substrate, over a range of pHs and temperatures, are shown in Fig. 7. The specific activity of the enzyme increased from 45 to 70 °C and declined thereafter. But the endoglucanase or endoglucanase activity sustained up to 80 °C. Similarly, in the case of pH, the specific activity was maximum in Na-acetate buffer with pH 4.5 followed by pH 7.0 and pH 8.0 compared to minimal activity at pH 3.0 as well as at pH 9.5. There was a sharp linear decline in endoglucanase activity beyond pH 7 and 76% of activity is retained at pH 8.5. In general, Tris and ammonia buffers with pH 8.5 and 9.5 were found to inhibit endoglucanase activity of *B. licheniformis* KBFB3.

#### Effect of metal ions on endoglucanase activity

**Fig. 8:**
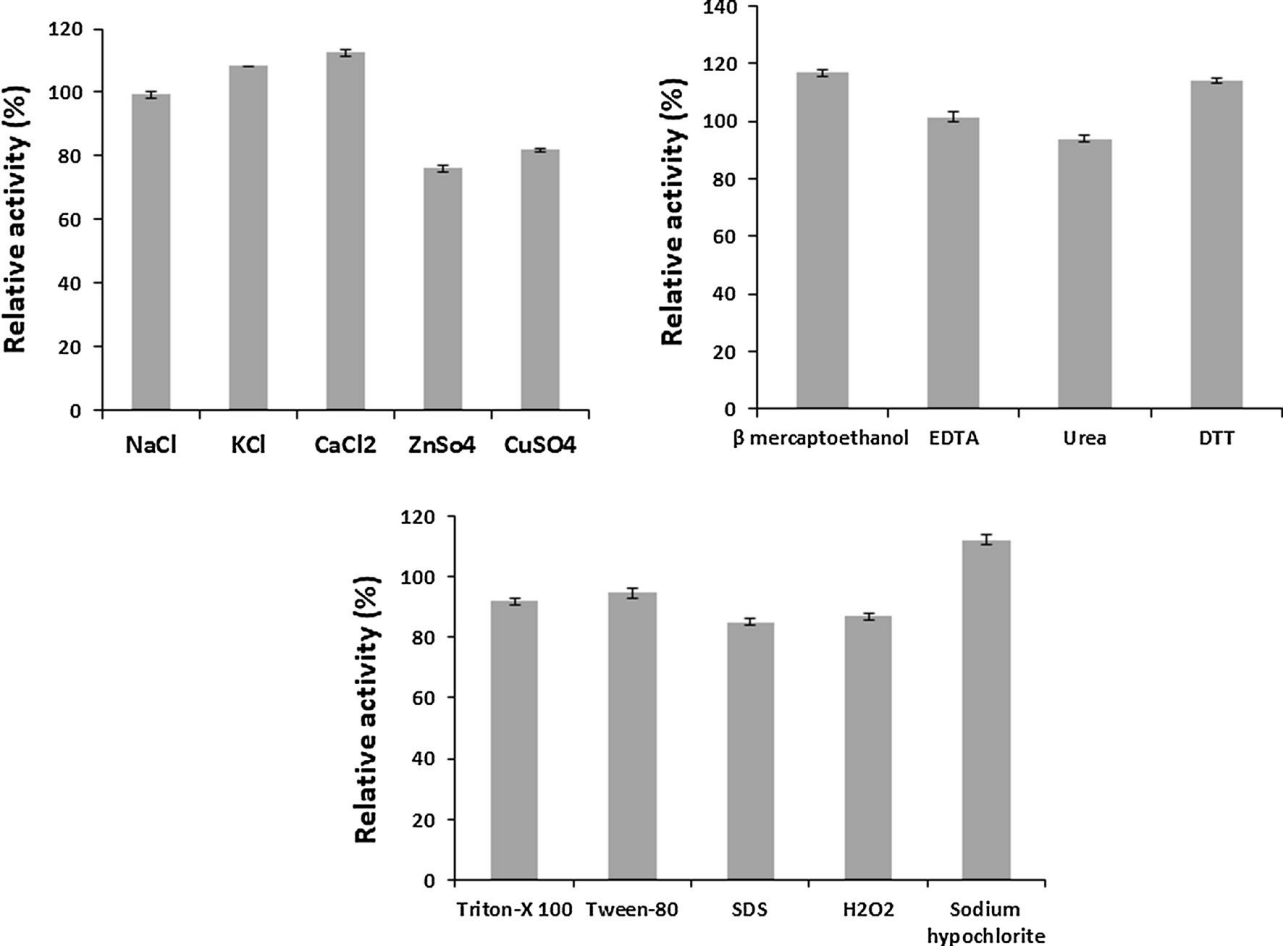
Effect of metal ions, inhibitors, surfactants, detergents and oxidizing agents on endoglucanase activity. Endoglucanase activity was determined at 70 °C at pH 7.0 in the presence of various metal ions, inhibitors, surfactants, detergents and oxidizing agents. After a period of 1 h pre-incubation, endoglucanase activity was assayed. The enzyme activity without pre-incubation with metal ions, inhibitors, surfactants, detergents and oxidizing agents was taken as 100%. Mean standard deviation for all the values is ± 5.0%

*Bacillus licheniformis* KBFB3 endoglucanase was activated by 5 mM Ca^2+^ and K^1+^ but inhibited by all other metal ions to a significant level. The results showed that endoglucanase exhibited maximum relative activity (108.2 and 112.4% in the presence of calcium and potassium ions whereas the relative activity with Na^1+^ was almost on par with K^1+^ ions. Endoglucanase activity was inhibited by Zn^2+^ and Cu^2+^ which registered 76 and 82% relative activity (Fig. 8).

#### Effect of inhibitors on endoglucanase activity

The effect of various inhibitors on endoglucanase stability was also investigated and the results are depicted in Fig. 8. The relative activity with 5 mM EDTA, β-mercaptoethanol, and DTT was retained at 117.0, 101.4 and 114.0% respectively whereas urea slightly inhibited the activity (94%).

#### Effect of surfactants, detergents and oxidizing agents on endoglucanase activity

The enzyme was extraordinarily stable in the presence of non-ionic surfactants like triton 100 and tween-80 with the relative activity of 92 and 94.7%, respectively (Fig. 8). Also, the detergent SDS inhibited the activity to a tune of 15% and retained the activity at 85% against control. Among the two oxidizing agents experimented, the endoglucanase activity was activated in the presence of sodium hypochlorite and H_2_O_2_ with relative activities 112.4 and 86.9% at 0.5% concentration.

## Discussion

Cellulase producing microbes are largely isolated from adverse environments and the majority of them are found to elaborate alkaline hydrolytic extracellular enzymes. However, so far only a few kinds of microbes with multifunctional enzyme complex have been reported (Zhang and Lynd 2004). Exploring lignocellulose rich samples, in which natural thermophilic biomass deconstruction occurs, might serve as a fruitful source for isolation of new thermophilic cellulase and xylanase producing candidates. Earlier studies adopted to switch grass and employed substrate specific enrichment technique for isolation of cellulose and hemicelluloses degrading bacteria (Eichorst et al. 2014). In the present study, thermophilic bacteria were isolated from the lignocellulosic rich substrates viz, pine needles, PABA, vanillin, acid extracted lignin, lignin extracted from black liquor, banana fibre, grapes, paddy straw, crop residue, corncob and saw dust which contained 25–50% cellulose, 37–50% hemicelluloses and 5–15% lignin. The enrichment of the lignocellulosic substrates at thermophilic hot springs yielded 38 thermophilic potential bacterial isolates that produce hydrolytic enzymes. Among the hot springs selected for study, Manikaran registered 65.79% of total thermophiles followed by Kalath (23.68%) and Vasist (10.53%). Among the 38 thermophilic bacteria isolated, 63.15% (24 isolates) of total bacteria exhibited cellulase cum xylanase activities while 20.83% of bacterial isolates showed only cellulase activity. These results clearly indicate a rich diversity of thermophilic bacteria producing glycosyl hydrolases type of enzymes in hot springs of Himachal Pradesh.

*Bacillus* sp. is gram-positive bacteria with a high level of extra cellular enzyme production capacity which has attracted its application in many industries. Cellulase and xylanase enzyme production by several *Bacillus* species has been reported by several workers (Heck et al. 2002; Bhalla et al. 2013; Sharma et al. 2015). Several cellulases with optimum activity at 40 °C have been identified, while the enzymatic hydrolysis at ≤ 50 °C exhibit slower hydrolysis rates and often incomplete. This problem can be overcome by thermostable enzymes isolated from thermophilic microbes growing at 50–80 °C. In this study, five cellulase cum xylanase producing thermophilic bacterial isolates namely *B. tequilensis* (VCB 1, VCB2 and VSDB4) and *B. licheniformis* (KBFB2 and KBFB3) were isolated from different cellulose and hemicelluloses rich substrates, banana fibre, saw dust and corn cob and selected based on preliminary plate assay. Maximum endo and exoglucanase activity were obtained in *B. licheniformis* KBFB3 (4.81 IU ml^−1^) and *B. tequilensis* VSDB4 (0.061 IU ml^−1^) isolated respectively from banana fibre and sawdust enriched sample, but low in *B. tequilensis* VCB2, isolated from corncob enrichment. Optimal cellulase activities at pH 6 after incubation at 40 °C were reported in *Bacillus circulans* and *Bacillus subtilis* (Otajevwo and Aluyi 2011). Thermostable endoglucanase activity was reported in *Bacillus amyloliquefaciens* DL-3 which could maintain 40% of its activity at broad pHs ranging from pH 4.0 to pH 9.0 after 20 h incubation at 50 °C (Lee et al. 2008; Sharma et al. 2015).

On the other hand, maximum FP activity (3.38 IU ml^−1^) was observed at 48 h in *B. tequilensis* VCB1 isolated by corn cob enrichment from Vasist. Similarly, *B. licheniformis* KBFB3 also showed maximum cellobiase activity. It was observed that *B. licheniformis* KBFB3 isolated from the banana fibre is endowed with multifunctional GH enzymes which could be attributed to the nature of substrates enriched. Even though the *B. licheniformis* KBFB2 was same as that of *B. licheniformis* KBFB3, both differed entirely in their GH profile. While *B. tequilensis* VSDB4 recorded good exoglucanase activity, *B. tequilensis* VCB1 had maximum FP activity. In this case, the substrate might have played a major role in determining the activity of a particular class of GHs. Natural fibers are generally hydrophilic in nature as they are derived from lignocellulose-containing strongly polarized hydroxyl groups (Zhang and Lynd 2004), and the high cellulose (64%) hemicellulose and lignin contents in the banana fibre attributes to efficient microbes with multiple GH functions (Eichorst et al. 2014).

Interestingly, the *Bacillus* species identified were able to produce cellobiase activity (aryl β-glucosidase) when they were grown in CMC (carboxy methyl cellulose). Thus, it was noted that *B. licheniformis* KBFB3 and *B. licheniformis* KBFB2 showed remarkable cellobiase production of 0.705 and 0.562 IU ml^−1^ at 48 h. For cellulose-grown organisms, there was more aryl II β-glucosidase than aryl III β-glucosidase. With growth on cellobiose, aryl III β-glucosidase increased and it did not hydrolyze carboxymethyl cellulase while hydrolyzing cellobiose. Thus it seems that during growth on a particular substrate (cellulose or cellobiose) those enzymes that are necessary for its breakdown are located more externally. Endoglucanase was more external in *Bacillus* sp. growing on the cellulosic substrate than those growing on cellobiose. This is in accordance with the studies of Ramasamy and Verachtert (1980) while localizing the cellulase components in *Pseudomonas* sp. The cell-bound aryl II β-glucosidases of *B. licheniformis* KBFB3 and *B. licheniformis* KBFB2 has to be elucidated. Prolonged incubation after 48 h brought about a decline in the enzyme activity and biomass conversion which might probably due to the consequence of random lethal events, including cellular fragmentation in the death phase and release of intracellular protease into the fermentation broth (Papagianni et al. 2002).

The PCR amplicons of Endoglucanase (CMCase) region (*CelS*) of about 250 bp for *B. tequilensis* (VCB1, VCB2 and VSDB4) and *Bacillus subtilis* VCB4 which served as positive control and 1300 bp for *B. licheniformis* (KBFB2 and KBFB3) further confirms the presence of cellulase encoding operons in all the isolates. The presence of cellulose binding domain was further evident by the amplification of *celB* gene (650 bp) in *B. licheniformis* KBFB2 and KBFB3. Using Ba_EN1 primer, amplification of about 250 bp cellulase genes in *B. subtilis* and cellulose binding domain of 650 bp fragments in *Bacillus* sp. was reported earlier by Bischoff et al. (2006) and Hussain et al. (2011) respectively.

Further characterization of partially purified endoglucanases (CMCase) from the thermophilic *Bacillus* sp. on SDS PAGE revealed 69 and 15 kDa fragments which were confirmed later by activity staining. This is compatible with the size of EGA reported by *Bacillus* sp Ac-1 (67 K Da) by Li et al. (2006, 2008). The multiple banding patterns observed with EGA might be due to cytoplasmic endoglucanases, periplasmic endoglucanases or aryl-β glucosidase II. In the present studies, endoglucanases were extracellular which is testified by the hydrolytic activity in agarose wells with CMC. Thus for growth on cellulose, the cell bound endoglucanase might have been more extracellular in localization.

It is evident from the kinetics study that the reaction rate (v) increases as the substrate [S] increases. However as [S] gets higher, the enzyme becomes saturated with the substrate and the rate reaches V_max_, the enzymes maximum rate. The isolate *B. licheniformis* KBFB3 had highest V_max_ and K_m_ values of 19.73 µmol min^−1^ and 111.5 m Mol to respectively for substrate concentration of 4% or 150 mM. Higher Km values reflect lower affinity between substrate and enzyme, indicating that the endoglucanase secreted by *B. licheniformis* KBFB3 had lower affinity for CMC than the *B. tequilensis* VSDB4 enzyme. In this study, the K_m_ for CMC was determined by assaying the endoglucanases using a cell-free culture supernatant which was partially purified with 70 to 80% ammonium sulfate. The literature suggests that the kinetic behaviour of cellulases is affected in the presence of other proteins (or substances) in the medium (Cascalheira and Queiroz 1999).

Partially purified endoglucanase obtained from the culture supernatant of promising elite isolate, *B. licheiformis* KBFB3 showed maximum specific activity (13.0 IU mg^−1^) compared to that reported by Li et al. (2006) and Singh and Kaur (2012) which was slightly less than the specific activity of purified enzyme activity obtained from *Bacillus* sp. However, further purification by ion exchange and gel filtration chromatographic would improve the specific activity of *B. licheiformis* KBFB3 as mentioned in previous reports. Characterization of endoglucanase from *B. licheniformis* KBFB3 showed that the optimal pH range is 4.5–7.0 and 70 °C, and the enzyme specific activity indicated a steady linear decrease thereafter. Even at alkaline pH of 8.5, endoglucanase of KBFB3 retained about 76% of residual specific activity. The thermal stability curves indicate that the enzyme was stable at 70 °C, which remained sustained till 80 °C, though declined sharply thereafter. *B. licheniformis* KBFB3 retained its specific activity up to pH 8.5 although for the other reported bacterial cellulases, the optimum pH of the crude cellulase was pH 5.0 (Mawadza et al. 2000). This observation indicates and endorses the thermo- and alkaline tolerant nature of endoglucanase of *B. licheniformis* KBFB3.

Thermal and alkaline/pH stability is another additional important characteristic of industrial enzymes. Thermophilic cellulases are required for many industrial applications, and only a few reports on thermophilic cellulases from mesophilic organisms are available (Li et al. 2006; Sangeeta et al., 2013; Bischoff et al. 2006). Endoglucanase from *Bacillus* sp. DUSEL R7 was active at 75 °C and has a half-life of 552 h at 60 °C (Rastogi et al. 2010). The mining of efficient thermotolerant Glycosyl hydrolases yielded a multifarious thermophilic isolate *B. licheniformis* KBFB3 with superior endoglucanases, beta-glucosidases and xylanases (data not shown). The hyper xylanase activity of *B. licheniformis* KBFB3 has not been discussed here and evaluation of its saccharification potential on different substrates is underway. Results of the present study evidence the suitability of the thermostable and alkalitolerant endoglucanases of *B. licheniformis* KBFB3 for possible efficient application in enzymatic hydrolysis of various lignocellulosic biomasses.

Endoglucanase activity was activated by 5 mM Ca^2+^ and K^1+^ ions and Na^1+^ has no adverse effect. Gaur and Tiwari (2015) had also reported that Ca^2+^, Na^1+^ and Mg ^2+^ ions strongly stimulated cellulase activity. Endoglucanase activity was inhibited by Zn^2+^ and Cu^2+^ metal ions. Similar results were reported for *Bacillus amyloliquefaciens* DL-3 (Lee et al. 2008) and *Bacillus vallismortis* RG-07 (Gaur and Tiwari 2015). The inhibition of endoglucanase activity by Zn^2+^ and Cu^2+^ ions could be due to decreased metalloenzyme activity resulted in the competition between exogenous cations and protein associated cations.

The endoglucanase of *Bacillus licheniformis* KBFB3 is stable in the presence of EDTA, β-mercaptoethanol, and DTT at a concentration of 5 mM. Similarly, Gaur and Tiwari (2015) reported that *Bacillus vallismortis* RG-07 retained activity with 10 mM EDTA, β-mercaptoethanol and DTT. The inhibition of endoglucanase activity by urea might be due to a slight degree of interaction with the SH group. However, DTT can reduce the disulfide bonds and re-nature their activity. The active site of endoglucanase of *B. licheniformis* KBFB3 may contain –SH group as reported by Gaur and Tiwari (2015) in *Bacillus vallismortis* RG-07. Wang et al. (2008) have reported that SDS and tween-80 moderately inhibited cellulase activity whereas Gaur and Tiwari (2015) observed that cellulase of *Bacillus vallismortis* RG-07 was highly stable with 1%SDS retaining 95% of activity. In the present study also the endoglucanase of *B. licheniformis* KBFB3 is moderately resistant to SDS at 0.5% and the effects of other detergents need to be elucidated.

Furthermore, the endoglucanase activity was substantially stable with oxidizing agents at a concentration of 0.5%. Similarly, Gaur and Tiwari (2015) reported that higher concentrations of oxidizing agents (1.0%) decreased the stability of cellulase except for sodium hypochlorite whereas lower concentrations (0.1%) enhanced the activity. This suggests that the endoglucanase of *B. licheniformis* KBFB3 is moderately tolerant to oxidizing agents and detergents and prove its potential applications in detergent formulations.

In spite of the fact that cellulase and xylanase are widely isolated from several organisms, it is the bacteria with higher growth rate produce enzyme complements which are stable at extreme temperature and pH. This particular feature gives an added advantage of utilizing bacterial cellulase and xylanase in simultaneous saccharification and fermentation process of waste recycling and biofuel production. The study further opens of new horizons of knowledge to look beyond unifunctional enzymes to multifunctional thermostable glycosyl hydrolases which have immense implications in biomass valorization to abate global energy crisis and environmental conservation. Nevertheless, research is underway worldwide to optimize cellulase production, which should facilitate efforts in the development of a robust and cost-effective process for the bioprocessing of cellulosic biomass to biofuels and value-added bioproducts. The present study has contributed significantly to the enhancement of existing knowledge on multifunctional biocatalysts from thermophilic bacteria from hot springs and holds the key for future development of ideal thermostable enzymes for industrial applications particularly valorization of biomass.

## Additional files

**Additional file 1: Table S1.** Gene specific primers for screening various Glycosyl hydrolases. **Table S2.** Kinetics of EGA as predicted by Michaelis–Menten model. **Table S3.** Summary of purification of Endoglucanase from *B. licheniformis* KBFB3.

**Additional file 2: Fig. S1.** Kinetics of endoglucanase (activity on different concentrations of substrate (CMC). The Km and V_max_ values for the five isolates for endoglucanase were calculated by Michelis Menten plots.
