# Supplementary material for: Bioprospecting thermophilic glycosyl hydrolases, from hot springs of Himachal Pradesh, for biomass valorization
Source: AMB Express. 2018 Oct 15;8:168. doi: 10.1186/s13568-018-0690-4 (PMC6188974; doi:10.1186/s13568-018-0690-4)
Supplement: Supplementary file 1 — Additional file 1: Table S1. Gene specific primers for screening various Glycosyl hydrolases. Table S2. Kinetics of EGA as predicted by Michaelis–Menten model. Table S3. Summary of purification of Endoglucanase from B. licheniformis KBFB3. [file 13568_2018_690_MOESM1_ESM.doc]

**Additional Material (AMBE-D-18-00082)**

**Journal Name: AMB Express**

**Bioprospecting thermophilic glycosyl hydrolases, from hot springs of Himachal Pradesh, for biomass valorization**

### *Sugitha Thankappan, Sujatha Kandasamy, Beslin Joshi, Ksenia N. Sorokina, Oxana P.Taran,and SivakumarUthandi**

**Department of Agricultural Microbiology, Tamil Nadu Agricultural University, Coimbatore-641003*

*^#^Boreskov Institute of catalysis (BIC), Siberian Branch of the Russian Academy of Sciences,*

*Novosibirsk, Russia.*

Corresponding author: [usivakumartnau@gmail.com](mailto:usivakumartnau@gmail.com); + 91-8903611294; Fax: 0422-6611294

Table S1. Gene specific primers for screening various Glycosyl hydrolases

| **Primer name** | **Primer sequences** | **References** |
| --- | --- | --- |
| CelS F  CelS R | 5’-GCCCTTGGTGTCCTTGATC 3’  5’- CTCTACAACAACATCTGGGG 3’ | Bischoff *et al*., 2006  Saarilhati*et al.,* 1990 |
| CelB F  CelB R | 5’-GTCCAGAACAACGCTGGGG -3’  5’-CGTTCTGCCACGGCTCGA-3’ |
| Ba_Xln F  Ba_XlnR | 5’- ATGTTTAAGTTTAAAAAGAATTTC-3’  5’- TTACCACACTGTTACGTTAG- 3’ | Hussain*et al*., 2011 |

**Table S2: Kinetics of EGA as predicted by Michaelis-Menten model**

| Best-fit values | VCB1 | VCB2 | VSDB4 | KBFB2 | KBFB3 |
| --- | --- | --- | --- | --- | --- |
| V_max_ | 5.367 | 5.345 | 8.453 | 19.73 | 11.62 |
| Km | 100.5 | 63.58 | 44.20 | 103.6 | 111.5 |

***Table S3: Summary of purification of Endoglucanase from B.licheniformis KBFB3***

| Purification Step | Total protein (mg) | Total activity (U) | Specific activity U (mg protein)^-1^ | Purification factor | Yield (%) |
| --- | --- | --- | --- | --- | --- |
| Culture supernatant | 0.61 | 3.38 | 5.54 | 1 | 100 |
| Ammonium sulfate fractionation | 0.37 | 4.81 | 13.00 | 2.34 | 142.2 |
