# Supplementary figures and images for: Bioprospecting thermophilic glycosyl hydrolases, from hot springs of Himachal Pradesh, for biomass valorization
Source: AMB Express. 2018 Oct 15;8:168. doi: 10.1186/s13568-018-0690-4 (PMC6188974; doi:10.1186/s13568-018-0690-4)

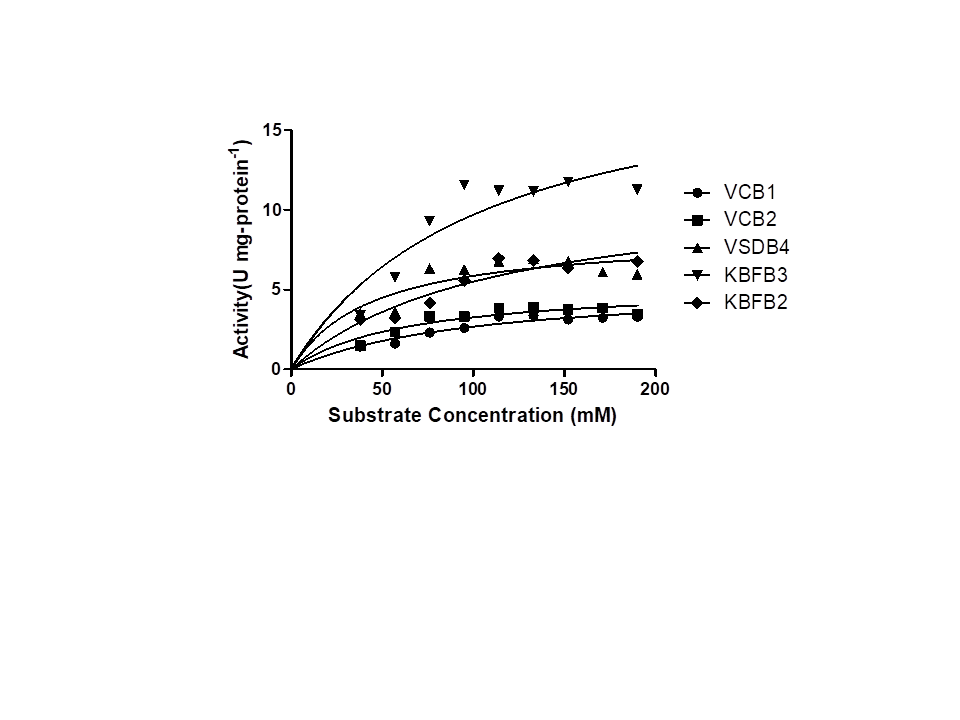

Supplement: Supplementary file 2 — Additional file 2: Fig. S1. Kinetics of endoglucanase (activity on different concentrations of substrate (CMC). The Km and Vmax values for the five isolates for endoglucanase were calculated by Michelis Menten plots. [file 13568_2018_690_MOESM2_ESM.tif]
